# Parasite clearance rates in Upper Myanmar indicate a distinctive artemisinin resistance phenotype: a therapeutic efficacy study

**DOI:** 10.1186/s12936-016-1240-7

**Published:** 2016-03-31

**Authors:** Kyaw Myo Tun, Atthanee Jeeyapant, Mallika Imwong, Min Thein, Sai Soe Moe Aung, Tin Maung Hlaing, Prayoon Yuentrakul, Cholrawee Promnarate, Mehul Dhorda, Charles J. Woodrow, Arjen M. Dondorp, Elizabeth A. Ashley, Frank M. Smithuis, Nicholas J. White, Nicholas P. J. Day

**Affiliations:** Defence Services Medical Research Centre, Naypyitaw, Myanmar; Myanmar Oxford Clinical Research Unit, Yangon, Myanmar; Mahidol-Oxford Tropical Medicine Research Unit, Faculty of Tropical Medicine, Mahidol University, 3rd Floor, 60th Anniversary Chalermprakiat Building, 420/6 Ratchawithi Rd., Ratchathewi District, Bangkok, 10400 Thailand; Department of Molecular Tropical Medicine and Genetics, Faculty of Tropical Medicine, Mahidol University, Bangkok, Thailand; Nuffield Department of Clinical Medicine, Centre for Tropical Medicine and Global Health, University of Oxford, Oxford, UK; Medical Action Myanmar, Yangon, Myanmar; Worldwide Antimalarial Resistance Network (WWARN), Bangkok, Thailand

**Keywords:** *Plasmodium falciparum*, Artemisinin resistance, Kelch 13 propeller, Parasite clearance half-life, Myanmar

## Abstract

**Background:**

Artemisinin resistance in *Plasmodium falciparum* extends across Southeast Asia where it is associated with worsening partner drug resistance and a decline in the efficacy of frontline artemisinin-based combination therapy. Dihydroartemisinin-piperaquine (DP) is an essential component of preventive and curative treatment in the region, but its therapeutic efficacy has fallen in Cambodia.

**Methods:**

A prospective clinical and parasitological evaluation of DP was conducted at two sites in Upper Myanmar between August 2013 and December 2014, enrolling 116 patients with acute uncomplicated falciparum malaria. Patients received DP orally for 3 days together with primaquine 0.25 mg/kg on admission. Parasite clearance half-lives based on 6 hourly blood smears, and day 42 therapeutic responses were assessed as well as parasite K13 genotypes.

**Results:**

Median parasite clearance half-life was prolonged, and clearance half-life was greater than 5 h in 21 % of patients. Delayed parasite clearance was significantly associated with mutations in the propeller region of the parasite *k13* gene. The *k13* F446I mutation was found in 25.4 % of infections and was associated with a median clearance half-life of 4.7 h compared with 2.7 h for infections without *k13* mutations (p < 0.001). There were no failures after 42 days of follow-up, although 18 % of patients had persistent parasitaemia on day 3.

**Conclusion:**

The dominant *k13* mutation observed in Upper Myanmar, F446I, appears to be associated with an intermediate rate of parasite clearance compared to other common mutations described elsewhere in the Greater Mekong Subregion. Discerning this phenotype requires relatively detailed clearance measurements, highlighting the importance of methodology in assessing artemisinin resistance.

**Electronic supplementary material:**

The online version of this article (doi:10.1186/s12936-016-1240-7) contains supplementary material, which is available to authorized users.

## Background

Artemisinin resistance in *Plasmodium falciparum* is now prevalent across much of mainland Southeast Asia [1–6]. Resistance is characterized by the clinical phenotype of delayed parasite clearance [7, 8] and is associated with mutations in the propeller region of the *P. falciparumkelch13* (*k13*) gene [6]. This marker has since been validated extensively in clinical studies and also by laboratory transfection [2–4, 9, 10]. Artemisinin resistance is clearly associated with ACT failures with reduced clinical efficacy of mefloquine-artesunate reported in Cambodia a decade ago [11, 12]. Four years after adoption of dihydroartemisinin-piperaquine (DP) in Cambodia, high failure rates were reported, with both artemisinin and piperaquine resistance as contributing factors [13–16]. Artemisinin resistance has steadily worsened along the Myanmar–Thailand border [17, 18] where the *k13* C580Y mutation now predominates, and is present in central and southern Myanmar where independent mutations have emerged [2, 3, 19]. A recent molecular survey of *k13* gene mutations, which obtained data from ten administrative regions, indicated that artemisinin resistance now extends across much of Myanmar [1].

The situation in relatively remote Northern Myanmar has been challenging to ascertain. Several clinical studies have been undertaken at the border between Kachin State and Yunnan Province in China [20, 21]; based on a combination of day 3 parasite positivity rates and subsequent molecular and in vitro studies [4, 5, 22] it has been concluded that artemisinin resistance is also present in this region.

Dihydroartemisinin-piperaquine is one of three artemisinin-based combinations registered in Myanmar, but it is not widely used because the main donors support distribution of artemether–lumefantrine free of charge through the government health system and international non-governmental organizations and at a reduced price in the private sector; nor was piperaquine monotherapy generally available in the past. Consistent with this there are no reports in Myanmar of reduced piperaquine sensitivity (in vivo or in vitro). DP is the only currently available drug for mass treatment in this region [23]. In order to investigate the degree and extent of artemisinin resistance in Northern and Central Myanmar, as well as the current efficacy of DP, we undertook a two-centre prospective study, using a combined protocol to characterize early parasitological responses (i.e. to assess artemisinin resistance) and the overall therapeutic efficacy of DP.

## Methods

### Study design

An open-label single arm clinical trial was conducted in two hospitals, one located in Myitkyina, Kachin State in Northern Myanmar and the other in Thabeikkyin, Mandalay Region in central Myanmar (Additional file 4: Figure S1). The two sites are situated in areas of low malaria transmission (entomological inoculation rate; EIR < 1) [24]. This trial was registered at ClinicalTrial.gov (NCT01350856). The Oxford Tropical Research Ethics Committee (UK) (OXTREC reference 06–11) and the Defence Services Medical Ethics Committee (Myanmar) approved the study protocol.

### Study participants

Inclusion criteria were acute uncomplicated *P. falciparum* malaria confirmed by positive blood smear on microscopy with asexual forms of *P. falciparum* (including mixed infection with non-falciparum species), parasitaemia of between 5000 and 200,000 per µL determined on a thin or thick blood film, and fever defined as >37.5 °C tympanic temperature or a history of fever. Male and non-pregnant female patients aged between 6 months and 65 years were recruited; children less than 5 years of age were not included at the Thabeikkyin site because of the absence of a paediatric specialist. Patients (or parents/guardians of minors) provided written informed consent to enrol in the study; those unable to read or write provided informed consent with a witness present. In patients from 10 to 17 years of age assent was also obtained. Exclusion criteria were signs of severe and/or complicated malaria [25], haematocrit less than 25 % or haemoglobin less than 8 g/dL, acute illness other than malaria requiring treatment, administration of artemisinin derivatives within the previous 7 days, history of allergy to artemisinins or to dihydroartemisinin-piperaquine, and previous splenectomy.

### Anti-malarials

Dihydroartemisinin-piperaquine (DP) was purchased as Duo-Cotecxin^®^ from Beijing Holley-Cotec Pharmaceuticals. Primaquine (Remedica Pharmaceuticals) was provided by 3MDG. Patients received DP orally each day for 3 days, with a target daily dose of 2.4 mg/kg dihydroartemisinin and 18 mg/kg piperaquine [25]. All patients received primaquine 0.25 mg/kg single dose on day 0 after the first dose of DP. Dosing was by weight categories (See Additional file 1). If the patient vomited within 1/2 h of anti-malarial ingestion, the dose was repeated. If vomiting occurred between 1/2 and 1 h, half the dose was repeated.

### Admission procedures

Patients were admitted to the hospital for at least 3 days for supervised treatment and 6 hourly blood smears. Parasite densities were estimated by blood smear every 6 h up to 48 h and daily thereafter until two negative consecutive slides were observed [26]. EDTA whole blood was collected at hour 0 and packed red cells stored at −20 °C for later *k13* gene sequencing and genotyping in case of recurrence.

### Follow up assessments

Tympanic temperature and haematocrit were measured every 6 h at the same time as malaria smears were taken, up to 48 h and then every 12 h until discharge from hospital. After patients were discharged from hospital, follow up assessments were performed at days 5, 7, 9, 14, 21 for malaria blood smear and haematocrit. A dried blood spot on Whatman^®^ 3MM cellulose chromatography paper was collected on day 7 for piperaquine drug levels. Thereafter, patients were assessed weekly at the clinic and blood samples were taken for malaria smear and haematocrit until day 42. Patients unable to come to the clinic were visited by research staff at home.

The trial was monitored by the MORU Clinical Trial Support Group and data evaluated for compliance with the protocol and accuracy in relation to source documents.

### Microscopy quality control measures and analysis of parasite clearance half-life

Giemsa-stained blood smears were prepared at the study sites. Parasitaemia (parasites/µl) was calculated from the thin blood film as parasitized red cells per 1000 red blood cells × haematocrit × 125.6, or the thick film as parasitized red cells × 8000/number of leucocytes counted (usually 200). All blood smears were read by a trained laboratory technician and all h0 and h72 slides, plus all slides from selected subjects were rechecked by experienced microscopists in the Malaria Laboratory, MORU, Bangkok [2].

### Molecular studies

Blood samples were processed using standard techniques (See Additional file 2). After PCR amplification, the main part of the *k13* gene (amino acids 210 onwards, including the whole propeller region) was sequenced. K13 sequences for a subset of patients were also incorporated into a published country-wide molecular marker survey in Myanmar in 2015 [1].

### Statistical analysis

Sample size was based on WHO guidelines for single-arm efficacy studies [27]. A target sample size of 73 enables detection of a 5 % failure rate with 95 % confidence and 5 % precision for each site. An additional 10 % was included to allow for losses to follow up. Based on previous studies, this number would also enable distribution of parasite clearance half-lives to be described adequately. Data were entered into a web-based database, OpenClinica, version 3.0. Data cleaning and analysis were done by using STATA statistical software, version 13 (StataCorp) and GraphPad Prism software 6.0 (Graphpad Software Inc.). Parasite clearance half-lives were calculated by using the online Parasite Clearance Estimator tool from the Worldwide Antimalarial Resistance Network [28].

Fever clearance was defined as the time to the start of the first 24-h period during which the temperature remained below 37.5 °C. The gametocyte clearance time (GCT) was the interval from first detection to last detection of gametocytes in a peripheral blood smear. Person-gametocyte weeks (PGW) were calculated for each case as the number of weeks in which gametocytaemia was patent (excluding admission) divided by duration of follow-up, expressed per 1000 person-weeks. Estimated haemoglobin values based on haematocrit were used to define anaemia (See Additional file 3).

Continuous variables were presented as mean and standard deviation, or if the distribution was non-normal as median and interquartile range. Tests of association between two categorical variables were performed using the Chi squared test, and comparison of non-normally distributed continuous variables by Mann–Whitney U test.

Simple logistic regression was conducted for association between each potential risk factor and prolonged parasite clearance half-life, with multiple logistic regression to analyse resulting risk factors.

## Results

**Fig. 1:**
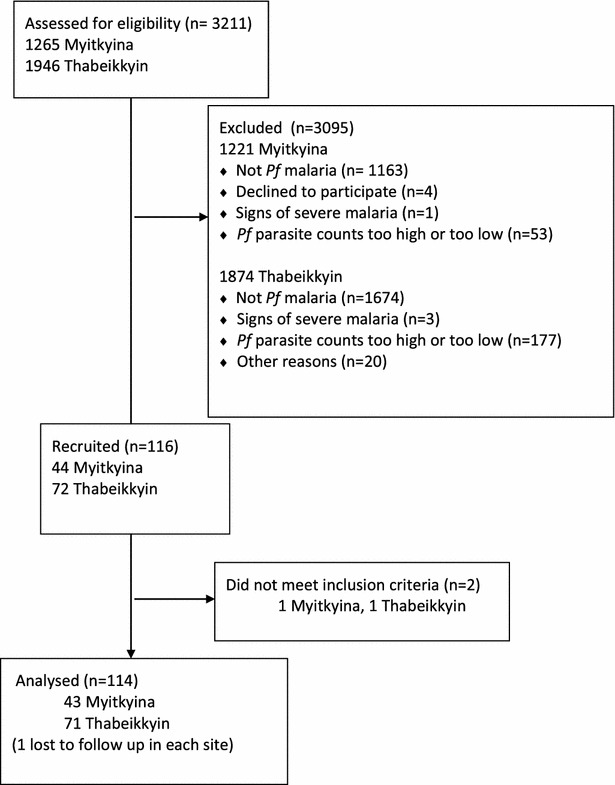
Participant flow chart

The study was conducted between August 2013 and December 2014 (See Additional file 4). A total of 3211 patients were screened and 116 patients enrolled, 44 in Myitkyina and 72 in Thabeikkyin (Fig. 1). Two patients were found not to meet the inclusion criteria after enrolment and were not analysed, leaving a total of 114 patients in the analysis. The aim was to recruit 80 patients per site but recruitment at the Myitkyina site was slower than anticipated, possibly owing to regional conflict.

### Baseline characteristics

Median patient age was 19 years (IQR 12–25), and males accounted for 75 % of all participants (Table 1). Geometric mean (95 % CI) parasite count on admission was 35,011 (28,613–42,839) parasites/µL with no significant difference between sites (p = 0.7). Mixed infection with *P. vivax* was present in 13 (11 %) of the 114 patients. Axillary temperature was >37.5 °C before treatment in a third of patients.

**Table 1:** Baseline characteristics of the patients by study site

Study site		Total	Myitkyina	Thabeikkyin
Number of patients		114	43	71
Gender (male sex)	n (%)	86 (75.4 %)	40 (93.0 %)	46 (64.8 %)
Age	year	19 (12–25)	23 (19–29)	13 (11–21)
Weight	kg	45 (30–54)	54 (45–56)	34 (23–50)
Height	m	1.57 (1.36–1.63)	1.63 (1.6–1.65)	1.47 (1.3–1.58)
BMI	kg/m^2^	18.3 (15.2–20.5)	20.5 (18.4–21.6)	16.3 (14.3–19.5)
Parasite count (geometric mean, 95 % CI)	No./µL	35,011 (28,613–42,839)	36,779 (26,809–50,446)	33,982 (26,016–44,287)
Gametocytaemia on day 0	n/N (%)	10/114 (8.8)	9/43 (20.9)	1/71 (1.4)
Mixed infection	n/N (%)	13/114 (11.4)	8/43 (18.6)	5/71 (7.1)
Haematocrit	%	37 (32–42)	38 (33–42)	37 (32–42)
Fever on admission	n/N (%)	37/114 (32.4)	12/43 (27.9)	25/71 (35.2)

Data are presented as median (IQR) unless otherwise indicated

### Clinical and early parasitological responses

**Fig. 2:**
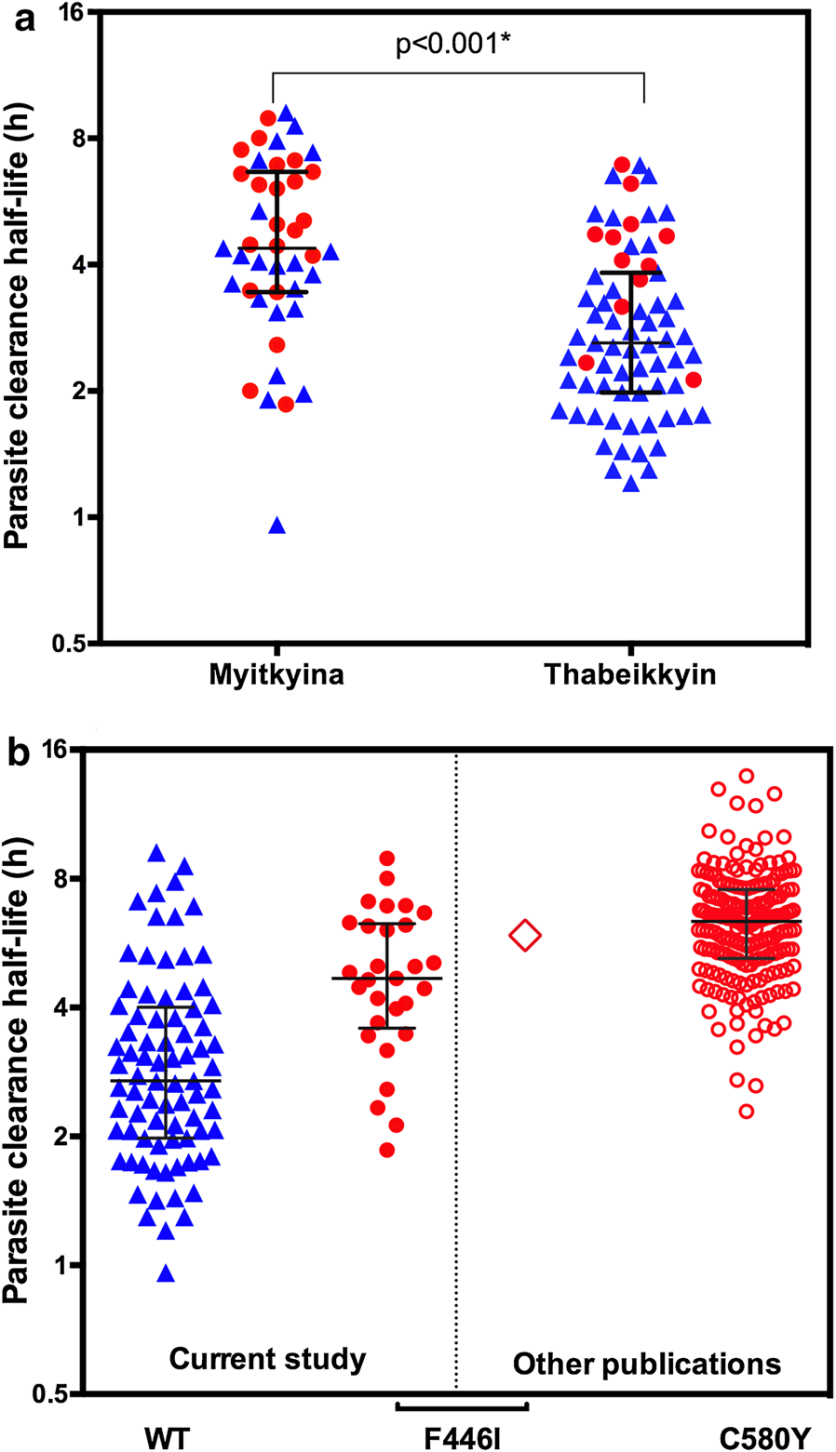
Distribution of parasite clearance half-lives by **a** geographical region **b** *Pf* K13 sequence. *Solid symbols* are from the current study, the *diamond* represents data from Huang et al. [4] and *hollow circles* are from Ashley et al. [2]. *Red circles* represent parasites with K13 propeller mutations, *blue triangles* represent wild-type. Median and IQR are shown

Median (IQR) parasite clearance half-life at Myitkyina was 4.4 h (3.4–6.7), significantly longer than Thabeikkyin [2.6 h (2.0–3.8, p < 0.001)] (Fig. 2a). In Myitkyina 39 % of patients had parasite clearance half-life more than 5 h compared with 13 % in Thabeikkyin (p = 0.001); using a 4 h cutoff these proportions were 63 and 23 %, respectively (p < 0.001).

Twenty of 114 participants (18 %) remained parasitaemic 3 days after treatment [30.2 % of patients in Myitkyina and 9.9 % in Thabeikkyin (p = 0.01)]. Fever resolution was rapid (median resolution time was 6 h in Myitkyina, 12 h in Thabeikkyin, p = 0.008) (Table 2).

**Table 2:** Clinical and parasitological responses

Study site	Total	Myitkyina	Thabeikkyin	P value
Median time of fever clearance (IQR), hours	6 (6–12)	6 (6–6)	12 (6–18)	0.008**
Median parasite clearance Half-life (IQR), hours	3.3 (2.1–4.8)	4.4 (3.4–6.7)	2.6 (2.0–3.8)	<0.0001**
Parasite clearance time (Median, IQR), hours	42 (30–72)	72 (42–96)	36 (24–72)	<0.0001**
Parasite clearance Half-life >4 h, n/N (%)	42/114 (37.7)	27/43 (62.8)	16/71 (22.5)	P < 0.001*
Parasite clearance half-life >5 h, n/N (%)	26/114 (22.8)	17/43 (39.5)	9/71 (12.7)	P < 0.01*
Day 3 parasitaemia, n/N (%)	20/114 (17.5)	13/43 (30.2)	7/71 (9.9)	P < 0.01*
Gametocyte carriage time (median, IQR), hours	18 (9–57)	57 (6–81)	16.5 (12–24)	0.31
ACPR on day 42, % (95 % CI)	100 (97–100)	100 (92–100)	100 (95–100)	

*ACPR* adequate clinical and parasitological response

* Chi square statistics

** Mann–Whitney test

### Curative efficacy

After initial parasite clearance all patients remained parasite free (including non-falciparum malaria) until 42 days (Table 2); two patients were lost to follow-up before 42 days with negative blood films at their last follow-up visit at 7 and 28 days.

### K13 sequence and parasitological response

The *k13* gene was successfully sequenced in all 114 samples. The prevalence of propeller (>440) mutations was 28.9 % (33/114) (Table 3); among these the F446I mutation predominated (29/33, 87.8 %). In Myitkyina 21 of 43 (49 %) of patients had *k13* mutations compared with 12 of 71 (16.9 %) in Thabeikkyin. All *k13* mutations observed had been reported in previous studies. No mutations were found in the non-propeller region of *k13* (amino acids 210–440).

**Table 3:** Prevalence of Pf K13 mutations

K13 genotype	Total n (%)	Myitkyina n (%)	Thabeikkyin n (%)
Wild type	81 (71.1)	22 (51.2)	59 (83.1)
P443S	1	1	–
F446I	29	18	11
G538 V	1	–	1
P574L	1	1	–
A676D	1	1	–
All K13 propeller mutations	33 (28.9)	21 (48.8)	12 (16.9)

Median parasite clearance half-life was 2.7 h for wild type *k13* and 4.7 h for F446I mutation (p < 0.001) (Fig. 2b). The single cases of propeller mutations P443S, G538V, P574L and A676D were associated with half-lives of 6.6, 4.7, 7.5, and 2.0 h, respectively. The proportion of patients still parasite positive at day 3 was higher in infections with parasites with *k13* mutations (10/33, 30.3 %) compared to those with wild type alleles (10/81, 12.3 %) (p = 0.02).

### Factors associated with prolonged parasite clearance

For multivariable analyses of factors associated with prolonged parasite clearance, half-life cut-off values of 4 and 5 h were used. With the 4 h cutoff, *k13* propeller mutation had an odds ratio of 5.0 (95 % CI 1.9–13.0, p = 0.001) while recruitment in Myitkyina had an odds ratio of 4.0 (1.7–10.1, p = 0.002). Using a cutoff of 5 h, these ratios were 2.3 (95 % CI 0.9–6.1, p = 0.1) and 3.5 (95 % CI 1.3–9.4, p = 0.01), respectively (Table 4).

**Table 4:** Multivariable analysis examining relationship between clearance half-life or day 3 positivity and covariates kelch 13 propeller mutations, site and admission parasitaemia

	Odds ratio	95 % confidence interval	P value
Half-life cutoff 4 h			
Site (Myitkyina)	4.1	1.7–10.1	0.002
Kelch 13 (>440)	5.0	1.9–12.9	0.001
Half-life cutoff 5 h			
Site (Myitkyina)	3.5	1.3–9.4	0.01
Kelch 13 (>440)	2.3	0.9–6.1	0.1
Day 3 positivity			
Parasitaemia on admission (>1 % parasitaemia)	4.0	1.2–11.4	0.02
Site (Myitkyina)	3.2	1.1–9.6	0.04
Kelch 13 (>440)	2.0	0.7–4.0	0.68

Persistent parasitaemia at day 3 was significantly associated with site and parasitaemia on admission (using a cutoff of 1 %, close to the median) (Table 4); in this multivariable analysis *k13* sequence was not a significant factor.

### Gametocytaemia

Ten patients (8.8 %) had patent gametocytaemia at the time of enrollment, with nine of these recruited in Myitkyina (Table 1). The geometric mean (95 % CI) gametocyte density on admission was 83/μL (34–201). Fourteen patients (12 %) developed patent gametocytaemia after admission. There was no association between gametocyte carriage and the presence of *k13* propeller mutations at the time of enrollment (p = 0.42) or after treatment (p = 1.0), suggesting that artemisinin resistance had no effect on the proportion of patients with gametocytes at or after admission.

The median (IQR) durations of gametocyte carriage in Myitkyina and Thabeikkyin were 57 (6–81) and 16.5 (12–24) h respectively. Overall gametocyte carriage rate was 26 PGW per 1000 weeks of follow up. There was no association between gametocyte carriage duration and geographical location (p = 0.31), or parasite clearance half-life (p = 0.25). All patients with gametocytaemia cleared gametocytes within 96 h from the time gametocytes were first seen.

### Haematological changes

**Fig. 3:**
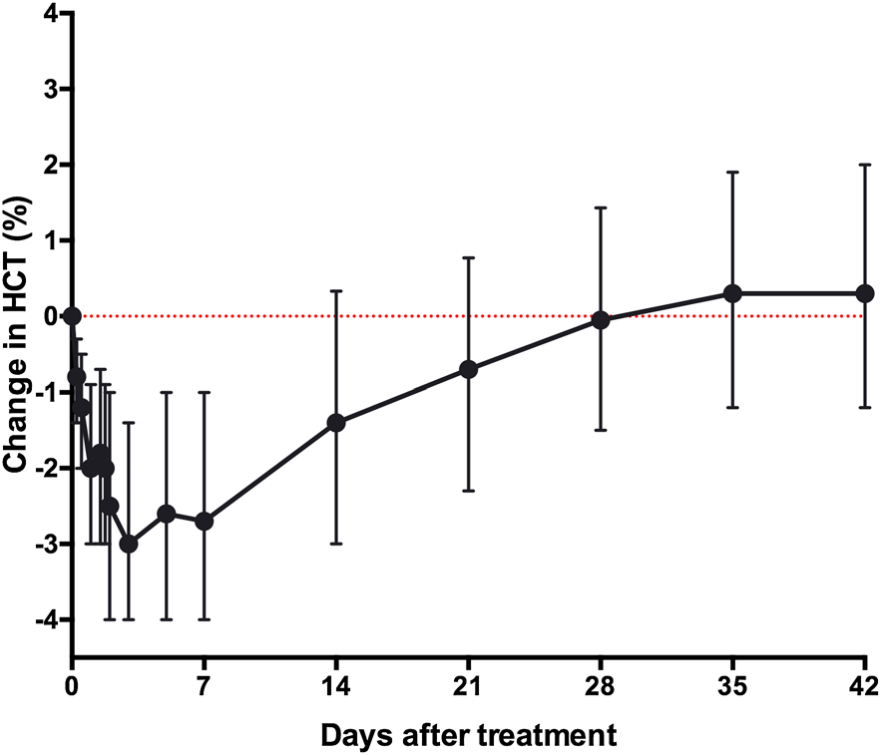
Mean (95 % CI) change in haematocrit compared with admission value during 42 day follow up

One third of participants (35 %) were anaemic on enrolment. The nadir in mean haematocrit occurred on day 3, after which haematocrit values gradually increased (Fig. 3; See Additional file 3). There were no clinically significant falls in haemoglobin, or haemoglobinuria. By day 28, most of the participants’ haematocrit values exceeded baseline measurements (Fig. 3).

### Safety and tolerance

The median dose (IQR) received by patients for dihydroartemisinin, piperaquine and primaquine were 2.2 (2.1–2.3), 17.5 (16.6–18.5) and 0.26 (0.24–0.28) mg/kg respectively. Study medications were generally well tolerated although six patients vomited the day 0 medication, and one vomited on day 2; all were retreated according to protocol. One serious adverse event was reported at the Myitkyina site; one patient who had already missed follow-up visits died in a road traffic accident approximately 2 weeks after the last visit.

## Discussion

In this study conducted in Northern and Central Myanmar DHA-piperaquine was highly efficacious, despite the presence of artemisinin resistance, with an overall day 3 positivity rate of 18 % (30 % in Myitkyina) [29]. The study protocol was designed specifically to assess prolongation of parasite clearance half-lives, the hallmark of artemisinin resistance in *P. falciparum* [29]. Parasite clearance half-life is less prone to confounding by baseline parasitaemia than day 3 parasite positivity [28, 30, 31], and remains the gold-standard for determining resistance phenotypes needed for the characterization of artemisinin resistance [10, 32–34]. The use of clearance half-life to define artemisinin resistance was validated in western Cambodia, where a dichotomous distribution of half-lives is evident and a 5-h cut-off provides satisfactory discrimination [30]. At the Myanmar-Thailand border where longitudinal studies have been undertaken for more than two decades, the C580Y mutation only recently replaced earlier K13 mutations associated with lesser effects on parasite clearance [2, 35].

Consistent with previous data from the Myanmar-China border and molecular surveys [1, 4, 5], F446I was the predominant *k13* mutation in this study. This mutation is associated with a significant slowing in parasite clearance, but these data indicate that this does not occur to the same extent as that associated with the mutations prevalent further east. The median parasite clearance half-life of F446I infections in this study was 4.7 h, less than that obtained by Huang et al. [4] in patients recruited nearby at the Myanmar–China border (less than 100 km from the Myitkyina site). This difference is most likely to reflect the more frequent measurement of parasitaemia in the current study; the protocol used 6-hourly assessment of parasitaemia in order to capture the lag phase of clearance and avoid systematic overestimation of slope half-life [26, 28]. Other factors might also have contributed to the differences, including host effects (fever, haematocrit, immune status and genetics [36]), drug factors, accuracy of microscopic assessment and stochastic effects due to sample size.

Until recently, there has been uncertainty with respect to the degree of artemisinin resistance in Northern Myanmar. The association of F446I, the prevalent *k13* mutation in the region, with an intermediate artemisinin resistance phenotype is consistent with clinical and laboratory studies undertaken at the Myanmar–China border. Several publications have documented relatively low day 3 parasite positivity rates in clinical studies from Yingjiang County, Yunnan Province [4, 20], where frequencies of the F446I mutation are generally 40–60 % [4, 5, 22]. For example, in the series of therapeutic efficacy studies from Yingjiang county described by Huang et al. [4], day 3 positivity rate was approximately 12 % while *k13* propeller mutation prevalence was 56 % (mostly F446I mutants). Most infections carrying *k13* propeller mutants were cleared by microscopy by day 3, suggesting the presence of a ‘milder’ resistance phenotype than observed in the TRAC (Tracking Resistance to Artemisinin) study (centred on western Cambodia) where most patients carrying *k13* mutations remained parasite positive at day 3 [2]. Two recently published studies from the China–Myanmar border have examined ring-stage survival in F446I parasites but these come to different conclusions with respect to the phenotypic effect [22, 37]. Overall, the F446I mutation prevalence has clearly risen over time at the Myanmar–China border [4, 5, 38], suggesting preferential survival after treatment compared to wild-type parasites, and thus selection.

In this study, site was also significantly associated with delayed parasite clearance (day 3 positivity and prolonged half-life), independent of *k13* mutations. Most probably, this could be a stochastic effect with long half-life infections simply representing the top end of the log-normal distribution of parasite half lives observed with *k13* wild-type infections [30]. This could also reflect host effects although an interesting, while an interesting although unlikely possibility is that artemisinin resistance might be mediated through an alternative (non-*k13*) mechanism. K13 wild-type infections remaining positive at day 3 have been found in other parts of Myanmar [39]. Genome-wide studies of parasite polymorphism will allow study of the population structure of F446I mutant parasites and determine whether underlying ‘backbone’ mutations play a role [32].

What is the future for DHA-piperaquine in Myanmar? At present, it remains a highly efficacious and well-tolerated treatment for falciparum malaria in Upper Myanmar, but if artemisinin resistance worsens, then increasing reliance will be placed on the piperaquine component. Worsening partner drug resistance has rapidly followed emergence of artemisinin resistance at both the Thai–Myanmar border [17] and in Cambodia, where piperaquine resistance has now emerged and DP failure rates have risen alarmingly in the last 2 years [14, 15, 40, 41].

The median (IQR) gametocyte carriage time in this study was 18 (9–57) h, significantly less than that in the TRAC study [160 (<6–252) h] where primaquine was not used [2]. Hence the addition of a single low dose of primaquine (0.25 mg/kg) resulted in low rates of gametocyte carriage and was well tolerated, with rapid haematological recovery from malarial anaemia. G6PD deficiency gene frequencies in Myanmar typically range from 10 to 20 %, so it is likely that G6PD-deficient male patients in this study were exposed to primaquine, but without significant haemolysis. This is in keeping with recent large-scale deployments in mass treatment where no serious adverse effects have been observed. Related to this, the patient population in Myitkina was predominantly young men (typically acquiring malaria because of military or mining activities in forested areas), an issue that would need to be taken into account in any systematic treatment programme.

In summary, this study shows that while treatment efficacy remains high with DHA-piperaquine, artemisinin resistance has emerged and is established in Upper Myanmar. However, the main *k13* mutation observed, *k13* F446I, was associated with a clearance half-life of slightly under 5 h, suggesting an “intermediate resistance” phenotype. The study also emphasizes the clearance half-life remains the gold-standard in research studies of artemisinin resistance and shows the value of frequent in vivo sampling of parasitaemia in locations where susceptibility to artemisinins is previously unknown [26]. The sampling schedule (6 hourly for 48 h then daily, until two negative slides) is more convenient for patients and investigators than that used in the TRAC study [2], but performs well [26].

## Additional files

10.1186/s12936-016-1240-7 Dosing Table. Dosing table of study drugs (Dihydroartemisinin-piperaquine and primaquine).
10.1186/s12936-016-1240-7 Sequencing of the *P. falciparum* kelch13 gene. Brief description of kelch13 gene sequencing and PCR primers.
10.1186/s12936-016-1240-7 Anaemia. Definition of anaemia and prevalence of anaemia in study population.
10.1186/s12936-016-1240-7 Additional figures. Geographic distribution of *k13* mutations in two study sites and study enrollment screening during study period.

## References

[1] Tun KM, Imwong M, Lwin KM, Win AA, Hlaing TM, Hlaing T (2015). Spread of artemisinin-resistant *Plasmodium falciparum* in Myanmar: a cross-sectional survey of the K13 molecular marker. Lancet Infect Dis.

[2] Ashley EA, Dhorda M, Fairhurst RM, Amaratunga C, Lim P, Suon S (2014). Spread of artemisinin resistance in *Plasmodium falciparum* malaria. N Engl J Med.

[3] Takala-Harrison S, Jacob CG, Arze C, Cummings MP, Silva JC, Dondorp AM (2015). Independent emergence of artemisinin resistance mutations among *Plasmodium falciparum* in Southeast Asia. J Infect Dis.

[4] Huang F, Takala-Harrison S, Jacob CG, Liu H, Sun X, Yang H (2015). A single mutation in K13 predominates in Southern China and is associated with delayed clearance of *Plasmodium falciparum* following artemisinin treatment. J Infect Dis.

[5] Wang Z, Shrestha S, Li X, Miao J, Yuan L, Cabrera M (2015). Prevalence of K13-propeller polymorphisms in *Plasmodium falciparum* from China–Myanmar border in 2007–2012. Malar J.

[6] Ariey F, Witkowski B, Amaratunga C, Beghain J, Langlois AC, Khim N (2014). A molecular marker of artemisinin-resistant *Plasmodium falciparum* malaria. Nature.

[7] Noedl H, Se Y, Sriwichai S, Schaecher K, Teja-Isavadharm P, Smith B (2010). Artemisinin resistance in Cambodia: a clinical trial designed to address an emerging problem in Southeast Asia. Clin Infect Dis.

[8] Dondorp AM, Nosten F, Yi P, Das D, Phyo AP, Tarning J (2009). Artemisinin resistance in *Plasmodium falciparum* malaria. N Engl J Med.

[9] Straimer J, Gnadig NF, Witkowski B, Amaratunga C, Duru V, Ramadani AP (2015). Drug resistance. K13-propeller mutations confer artemisinin resistance in *Plasmodium falciparum* clinical isolates. Science.

[10] Takala-Harrison S, Clark TG, Jacob CG, Cummings MP, Miotto O, Dondorp AM (2013). Genetic loci associated with delayed clearance of *Plasmodium falciparum* following artemisinin treatment in Southeast Asia. Proc Natl Acad Sci USA.

[11] Rogers WO, Sem R, Tero T, Chim P, Lim P, Muth S (2009). Failure of artesunate-mefloquine combination therapy for uncomplicated *Plasmodium falciparum* malaria in southern Cambodia. Malar J.

[12] Denis MB, Tsuyuoka R, Poravuth Y, Narann TS, Seila S, Lim C (2006). Surveillance of the efficacy of artesunate and mefloquine combination for the treatment of uncomplicated falciparum malaria in Cambodia. Trop Med Int Health.

[13] Leang R, Barrette A, Bouth DM, Ménard D, Abdur R, Duong S (2013). Efficacy of dihydroartemisinin-piperaquine for treatment of uncomplicated *Plasmodium falciparum* and *Plasmodium vivax* in Cambodia, 2008–2010. Antimicrob Agents Chemother.

[14] Spring MD, Lin JT, Manning JE, Vanachayangkul P, Somethy S, Bun R (2015). Dihydroartemisinin-piperaquine failure associated with a triple mutant including kelch13 C580Y in Cambodia: an observational cohort study. Lancet Infect Dis.

[15] Saunders DL, Vanachayangkul P, Lon C (2014). Dihydroartemisinin-piperaquine failure in Cambodia. N Engl J Med.

[16] Chaorattanakawee S, Saunders DL, Sea D, Chanarat N, Yingyuen K, Sundrakes S (2015). Ex vivo drug susceptibility testing and molecular profiling of clinical *Plasmodium falciparum* isolates from Cambodia from 2008 to 2013 suggest emerging piperaquine resistance. Antimicrob Agents Chemother.

[17] Carrara VI, Lwin KM, Phyo AP, Ashley E, Wiladphaingern J, Sriprawat K (2013). Malaria burden and artemisinin resistance in the mobile and migrant population on the Thai-Myanmar border, 1999–2011: an observational study. PLoS Med.

[18] Phyo AP, Nkhoma S, Stepniewska K, Ashley EA, Nair S, McGready R (2012). Emergence of artemisinin-resistant malaria on the western border of Thailand: a longitudinal study. Lancet.

[19] Kyaw MP, Nyunt MH, Chit K, Aye MM, Aye KH, Aye MM (2013). Reduced susceptibility of *Plasmodium falciparum* to artesunate in southern Myanmar. PLoS One.

[20] Wang Y, Yang Z, Yuan L, Zhou G, Parker D, Lee MC (2015). Clinical efficacy of dihydroartemisinin-piperaquine for the treatment of uncomplicated *Plasmodium falciparum* malaria at the China–Myanmar border. Am J Trop Med Hyg.

[21] Huang F, Tang L, Yang H, Zhou S, Sun X, Liu H (2012). Therapeutic efficacy of artesunate in the treatment of uncomplicated *Plasmodium falciparum* malaria and anti-malarial, drug-resistance marker polymorphisms in populations near the China–Myanmar border. Malar J.

[22] Wang Z, Wang Y, Cabrera M, Zhang Y, Gupta B, Wu Y (2015). Artemisinin resistance at the China-Myanmar border and association with mutations in the K13 propeller gene. Antimicrob Agents Chemother.

[23] WHO (2014). Minutes of the drug resistance and containment Technical Expert Group.

[24] Gething PW, Patil AP, Smith DL, Guerra CA, Elyazar IR, Johnston GL (2011). A new world malaria map: *Plasmodium falciparum* endemicity in 2010. Malar J.

[25] WHO (2015). Guidelines for the treatment of malaria.

[26] Flegg JA, Guerin PJ, Nosten F, Ashley EA, Phyo AP, Dondorp AM (2013). Optimal sampling designs for estimation of *Plasmodium falciparum* clearance rates in patients treated with artemisinin derivatives. Malar J.

[27] WHO (2009). Methods for surveillance of antimalarial drug efficacy.

[28] Flegg JA, Guerin PJ, White NJ, Stepniewska K (2011). Standardizing the measurement of parasite clearance in falciparum malaria: the parasite clearance estimator. Malar J.

[29] WHO (2015). Status report on artemisinin and ACT resistance.

[30] White LJ, Flegg JA, Phyo AP, Wiladpai-ngern JH, Bethell D, Plowe C (2015). Defining the in vivo phenotype of artemisinin-resistant falciparum malaria: a modelling approach. PLoS Med.

[31] Worldwide Antimalarial Resistance Network (2015). (WWARN). Baseline data of parasite clearance in patients with falciparum malaria treated with an artemisinin derivative: an individual patient data meta-analysis. Malar J.

[32] Miotto O, Amato R, Ashley EA, MacInnis B, Almagro-Garcia J, Amaratunga C (2015). Genetic architecture of artemisinin-resistant *Plasmodium falciparum*. Nat Genet.

[33] Cheeseman IH, Miller BA, Nair S, Nkhoma S, Tan A, Tan JC (2012). A major genome region underlying artemisinin resistance in malaria. Science.

[34] Stepniewska K, Ashley E, Lee SJ, Anstey N, Barnes KI, Binh TQ (2010). In vivo parasitological measures of artemisinin susceptibility. J Infect Dis.

[35] Anderson TJC, Nair S, McDew-White M, Cheeseman IH, Bilgic F, McGready R (2014). Anatomy of an ongoing soft selective sweep malaria parasites driven by artemisinin treatment.

[36] Amaratunga C, Sreng S, Suon S, Phelps ES, Stepniewska K, Lim P (2012). Artemisinin-resistant *Plasmodium falciparum* in Pursat province, western Cambodia: a parasite clearance rate study. Lancet Infect Dis.

[37] Ye R, Hu D, Zhang Y, Huang Y, Sun X, Wang J (2016). Distinctive origin of artemisinin-resistant *Plasmodium falciparum* on the China–Myanmar border. Sci Rep.

[38] Feng J, Zhou D, Lin Y, Xiao H, Yan H, Xia Z (2015). Amplification of pfmdr1, pfcrt, pvmdr1, and K13 propeller polymorphisms associated with *Plasmodium falciparum* and *Plasmodium vivax* isolates from the China–Myanmar border. Antimicrob Agents Chemother.

[39] Nyunt MH, Hlaing T, Oo HW, Tin-Oo LL, Phway HP, Wang B (2015). Molecular assessment of artemisinin resistance markers, polymorphisms in the k13 propeller, and a multidrug-resistance gene in the eastern and western border areas of Myanmar. Clin Infect Dis.

[40] Leang R, Taylor WR, Bouth DM, Song L, Tarning J, Char MC (2015). Evidence of *Plasmodium falciparum* malaria multidrug resistance to artemisinin and piperaquine in Western Cambodia: dihydroartemisinin-piperaquine open-label multicenter clinical assessment. Antimicrob Agents Chemother.

[41] Fairhurst RM (2015). High antimalarial efficacy of dihydroartemisinin-piperaquine on the China–Myanmar border: the calm before the storm. Am J Trop Med Hyg.

