# Supplementary material for: Parasite clearance rates in Upper Myanmar indicate a distinctive artemisinin resistance phenotype: a therapeutic efficacy study
Source: Malar J. 2016 Mar 31;15:185. doi: 10.1186/s12936-016-1240-7 (PMC4815199; doi:10.1186/s12936-016-1240-7)
Supplement: Supplementary file 1 — 10.1186/s12936-016-1240-7 Dosing Table. Dosing table of study drugs (Dihydroartemisinin-piperaquine and primaquine). [file 12936_2016_1240_MOESM1_ESM.docx]

**Additional file 1 Dosing table**

Table (A1) Dihydroartemisinin-piperaquine and Primaquine dosing table

| Weight (kg) | DP (40+320 mg) | | Primaquine (7.5 mg) | |
| --- | --- | --- | --- | --- |
|  | Tabs | (or mls )^1^ | Tabs | (or mls )^1^ |
| 5 |  | 1.3 |  | 0.8 |
| 6 |  | 1.6 |  | 1 |
| 7 |  | 2 |  | 1.2 |
| 8-12 | ½ |  | ¼ |  |
| 13-20 | 1 |  | ½ |  |
| 21-30 | 1 ½ |  | 1 |  |
| 31-40 | 2 |  | 1 ¼ |  |
| 41-50 | 2 ½ |  | 1½ |  |
| 51-60 | 3 |  | 2 |  |
| 61-70 | 3 ½ |  | 2¼ |  |
| 71-84 | 4 |  | 2½ |  |
| 85-100 | 5 |  | 3 |  |

1. Liquid form of DP or PQ can be obtained by crushing the tablet and mixing with 5 ml of drinking water.
