# Supplementary material for: Parasite clearance rates in Upper Myanmar indicate a distinctive artemisinin resistance phenotype: a therapeutic efficacy study
Source: Malar J. 2016 Mar 31;15:185. doi: 10.1186/s12936-016-1240-7 (PMC4815199; doi:10.1186/s12936-016-1240-7)
Supplement: Supplementary file 2 — 10.1186/s12936-016-1240-7 Sequencing of the P. falciparum kelch13 gene. Brief description of kelch13 gene sequencing and PCR primers. [file 12936_2016_1240_MOESM2_ESM.docx]

#### Additional file (A2)

#### Sequencing of the P. falciparum kelch13 gene (PF3D7_1343700)

Genomic DNA was extracted using QIAamp® DNA Mini Kit (QIAGEN, Germany), following the manufacturer’s instructions. The K13 gene (amino acids 210 onwards) was sequenced by standard dideoxy sequencing of PCR products. These were obtained by nested PCR, the initial PCR (nest 1) amplifying the whole gene (spanning 2438 bases) and two nested PCR reactions (nest 2, fragments b-c) each amplifying a fragment (approximately 850 – 950 bases) of the kelch13 gene. PCR reaction conditions consisted of a final reaction volume of 100μl containing 10mM Tris-HCl (pH 8.3), 50mM KCl, 2mM MgCl2 (3mM for fragment c), 125μM 4-deoxynucleotide triphosphate (dNTPs), 250nM oligonucleotide primers (see Appendix Table S1), 2μl of each genomic DNA template, and 0.4 units Platinum®Taq DNA polymerase (Invitrogen, USA). The cycling parameters were pre-denaturation 95°C for 5 min, followed by 25 (nest 1) or 35 (nest 2) PCR cycles involving denaturation at 94°C for 1 min, annealing at 58°C for 2 minutes and extension at 72°C for 2 minutes, with post-extension at 72°C for 7 min, using a MyCyclerTM thermal cycler (Bio-Rad Laboratories, U.S.A.). Purified PCR products were sequenced at Macrogen, Republic of Korea and analysed using ImageLab (BioRad Gel doc XR) with a reference of 3D7 kelch13 sequence (Accession: XM_001350122.1).

#### Recurrent infection

For recurrent infections, PCR parasite genotyping on the baseline and recurrent samples was performed for MSP1, MSP2, and GLURP to define reinfection or recrudescence [1].

Table (A2) PCR primers

| Reaction | Fragment | Primer name | Sequence(5’>3’) | Product(bp) |
| --- | --- | --- | --- | --- |
| Nest 1 | Whole gene | K13_c.-155F  K13_c.2283R | AACAAGGCGTAAATATTCGTGT  TGTGCATGAAAATAAATATTAAAGAAG | 2483 |
| Nest 2 | Fragment b | K13_c.614F  K13_c.1464R | TTGAAACGGAATTAAGTGATGC  CAATACAGCACTTCCAAAATAAGC | 851 |
| Nest 2 | Fragment c | K13_c.1344F  K13_c.2283R | AGGTGGATTTGATGGTGTAGAA  TGTGCATGAAAATAAATATTAAAGAAG | 940 |

Reference:

1. Snounou G, Zhu X, Siripoon N, Jarra W, Thaithong S, Brown KN, et al: Biased distribution of msp1 and msp2 allelic variants in Plasmodium falciparum populations in Thailand. *Trans R Soc Trop Med Hyg* 1999, 93:369-374.
