# Supplementary material for: Parasite clearance rates in Upper Myanmar indicate a distinctive artemisinin resistance phenotype: a therapeutic efficacy study
Source: Malar J. 2016 Mar 31;15:185. doi: 10.1186/s12936-016-1240-7 (PMC4815199; doi:10.1186/s12936-016-1240-7)
Supplement: Supplementary file 3 — 10.1186/s12936-016-1240-7 Anaemia. Definition of anaemia and prevalence of anaemia in study population. [file 12936_2016_1240_MOESM3_ESM.docx]

#### Additional file 3

#### Anaemia

In accordance with WHO criteria [1], anaemia was defined as haemoglobin less than 11 g/dL or less than 10 g/dL in children under five years of age. Estimated haemoglobin values were obtained from original haematocrit measurements using a published conversion method (haematocrit = 5.62 + 2.60 x haemoglobin) [2].

Table (A3) Prevalence of Anaemia during study period

| Site | Day0  n/N(%) | Day7  n/N(%) | Day14  n/N(%) | Day21  n/N(%) |
| --- | --- | --- | --- | --- |
| Myitkyina | 14/43 (32.5) | 12/43(27.9) | 10/43(23.2) | 11/43(25.5) |
| Thabeikkyin | 26/71(36.6) | 38/71(53.5) | 35/71(49.3) | 23/71(32.3) |
| Total | 40/114(35) | 50/114(43.8) | 45/114(39.4) | 34/114(29.8) |

Reference:

1. WHO: Haemoglobin concentrations for the diagnosis of anaemia and assessment of severity. In *Vitamin and Mineral Nutrition Information System*. Geneva: World Health Organization; 2011.

2. Lee SJ, Stepniewska K, Anstey N, Ashley E, Barnes K, Binh TQ, et al: The relationship between the haemoglobin concentration and the haematocrit in Plasmodium falciparum malaria. *Malar J* 2008, 7:149.
