# Supplementary material for: Parasite clearance rates in Upper Myanmar indicate a distinctive artemisinin resistance phenotype: a therapeutic efficacy study
Source: Malar J. 2016 Mar 31;15:185. doi: 10.1186/s12936-016-1240-7 (PMC4815199; doi:10.1186/s12936-016-1240-7)
Supplement: Supplementary file 4 — 10.1186/s12936-016-1240-7 Additional figures. Geographic distribution of k13 mutations in two study sites and study enrollment screening during study period. [file 12936_2016_1240_MOESM4_ESM.docx]

**Additional file 4 Additional figures**


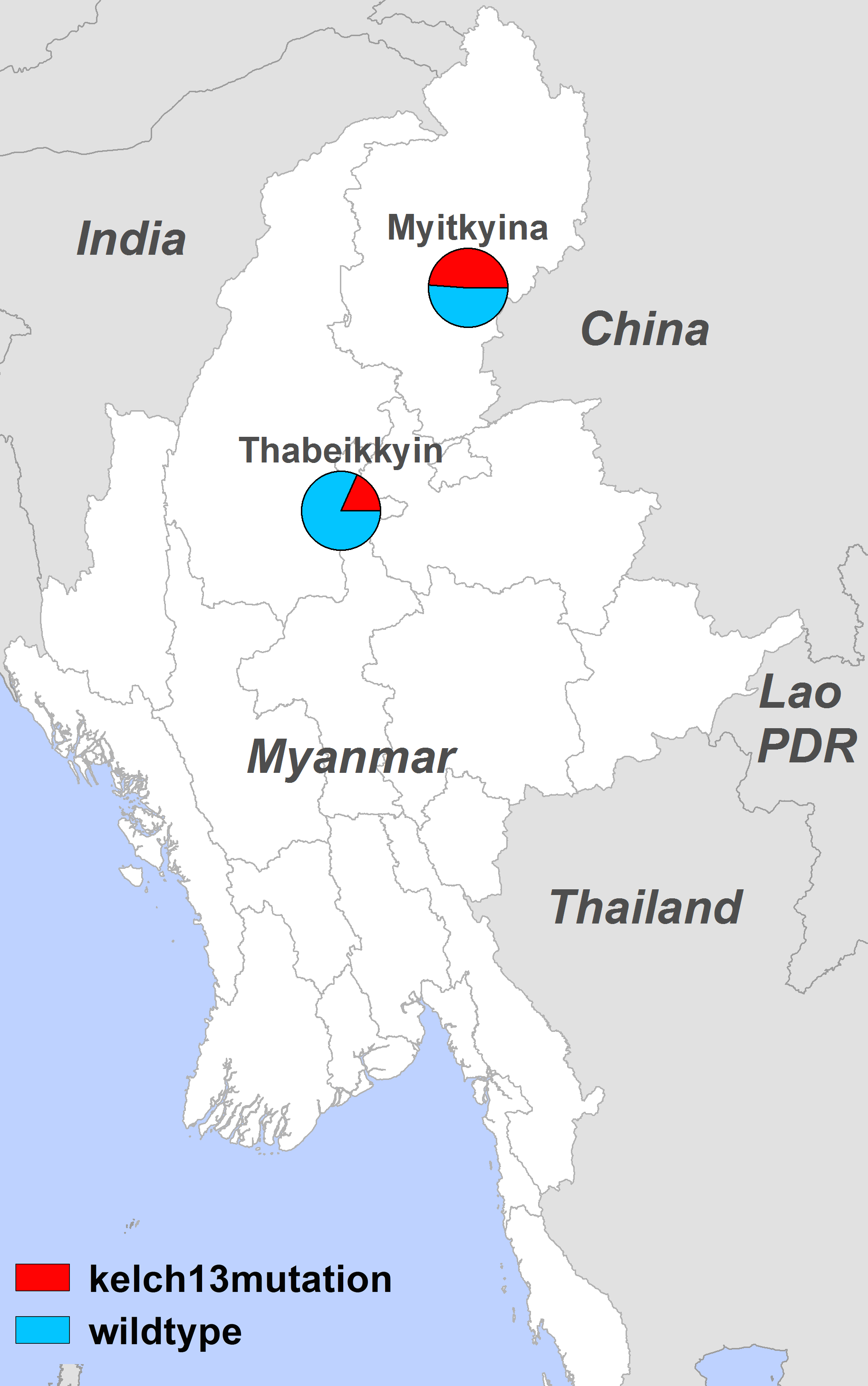


Figure (A1) Geographic distribution of *k13* mutations in two study sites of Myanmar

Figure (A2) Study enrollment screening from August 2013 to December2014
